# Presence of *Coxiella burnetii* DNA in inflamed bovine cardiac valves

**DOI:** 10.1186/s12917-017-0988-5

**Published:** 2017-03-09

**Authors:** Jørgen S. Agerholm, Tim K. Jensen, Jens F. Agger, Marc Y. Engelsma, Hendrik I. J. Roest

**Affiliations:** 1 Department of Large Animal Sciences, Faculty of Health and Medical Sciences, University of Copenhagen, Hojbakkegaard Alle 5, DK-2630 Taastrup, Denmark; 2 National Veterinary Institute, Technical University of Denmark, Bülowsvej 27, DK-1870 Frederiksberg C, Denmark; 3 Department of Diagnostics and Crisis Organisation, Wageningen Bioveterinary Research, Edelhertweg 15, 8219 PH Lelystad, The Netherlands; 4 Department of Bacteriology and Epidemiology, Wageningen Bioveterinary Research, Edelhertweg 15, 8219 PH Lelystad, The Netherlands

**Keywords:** Cattle, *Coxiella burnetii*, Endocarditis, PCR, Q fever

## Abstract

**Background:**

Bacterial endocarditis is a recognised disease in humans and animals. In humans, infection with *Coxiella burnetii* can cause endocarditis, but this has not been investigated thoroughly in animals. Endocarditis in cattle is a common post-mortem finding in abattoirs and studies have identified *Trueperella pyogenes* as a major cause. Despite exposure of cattle to *C. burnetii*, the significance of this particular bacterium for development and progression of endocarditis has not been studied in detail. Cardiac valves of cattle affected with endocarditis (*n* = 100) were examined by histology, fluorescence in situ hybridization (FISH) and real time quantitative polymerase chain reaction (PCR). Serum was examined for anti-*C. burnetii* antibodies by enzyme-linked immunosorbent assay (ELISA).

**Results:**

Serology revealed that 70% of the cattle were positive for antibodies to *C. burnetii*, while PCR analysis identified 25% of endocarditis valve samples as being positive. *C. burnetii* was not detected by FISH, probably due to the low infection levels. Most cattle had chronic valvular vegetative endocarditis with lesions being characterised by a core of fibrous tissue covered by significant amounts of fibrin, sometimes with areas of liquefaction, and with a coagulum covering the surface. In a few cases, including the case with the highest infection level, lesions were characterized by extensive fibrosis and calcification. Histologically, bacteria other than *C. burnetii* were observed in most cases.

**Conclusions:**

The presence of *C. burnetii* DNA is relatively common in cattle affected with valvular endocarditis. The role of *C. burnetii* remains however unknown as lesions did not differ between *C. burnetii* infected and non-infected cattle and because *T. pyogenes*–like bacteria were present in the inflamed valves; a bacterium able to induce the observed lesions. Heart valves of normal cattle should be investigated to assess if *C. burnetii* may be present without preexisting lesions.

## Background

*Coxiella burnetii* is a Gram-negative obligate intracellular bacterium that infects a wide range of mammalian species, and causes the disease syndrome Q fever. Human cases of Q fever are generally regarded as being associated with exposure to domestic ruminants although a significant proportion of cases do not report a direct contact to animals [1].

In humans, infection is either subclinical or results in a self-limiting febrile illness. The infection may become chronic and lead to development of endocarditis in those individuals with predisposing conditions, such as valvulopathy, prosthetic valve implants, vascular abnormalities or immunosuppression [2]. In addition, infection during pregnancy carries an increased risk of miscarriage [3–5].

The diagnosis of Q fever in animals is typically associated with abortion or delivery of weak or stillborn offspring. Such reproductive consequences occur sporadically in cattle, while flock outbreaks have been reported in goats and sheep [6, 7]. There is little in the way of published research into non-reproductive clinical manifestations of Q fever in ruminant species, despite the documented high seroprevalence against *C. burnetii* reported in livestock [8]. However, circulating *C. burnetii* DNA has been detected sporadically in blood of cattle thus indicating that some animals occasionally develop coxiellaemia [9]. Based on the comparative aspects in humans, where *C. burnetii* is a well-known cause of endocarditis, it could be suspected that *C. burnetii* may also be implicated in the development or progression of endocarditis in cattle under certain circumstances.

Valvular endocarditis is a well-recognised condition in cattle, with an estimated prevalence of 1–2% observed during post-mortem inspection at abattoirs [10, 11]. The aetiology has been investigated using routine microbiological techniques in several studies and the cultureable bacterial flora of bovine endocarditis is well-known. *Trueperella pyogenes*, a major opportunistic pathogen of cattle, has been identified in the majority of cases [12–16], although streptococci were reported in an older study [17]. However, some bacteria, such as *C. burnetii,* cannot be cultured by standard bacteriological methods as the bacterium requires cell cultures for propagation due to its intracellular nature. Studies specifically targeting *C. burnetii* in bovine endocarditis cases have not been done, but the hypothesis of *C. burnetii* being associated with endocarditis in animals has been tested in northern sea otters, which inhabit an environment where marine mammals are exposed to *C. burnetii*; however *C. burnetii* was not found in cases of endocarditis [18].

Danish dairy cattle are frequently seropositive for *C. burnetii* thus showing a widespread exposure to this bacterium [19]. As endocarditis is a common finding in Danish slaughter cattle as well [11] and as cattle is expected to experience episodes of coxiellaemia [9], we performed a study to investigate if *C. burnetii* could be detected in inflamed cardiac valves of Danish cattle.

## Methods

### Study population and samples

Cardiac valves and blood samples were obtained from cattle (*n* = 100), where there was evidence of endocarditis at post mortem inspection at three Danish abattoirs (Danish Crown Tonder, Holsted and Aalborg), during a 12 month period. The heart was incised during routine post-mortem examination and the endocardium inspected for lesions. If present, a representative tissue sample was taken from the inflamed valve and blood was obtained from the heart or large veins. The samples and information on the location of cardiac lesions and presence of other lesions were submitted to the University of Copenhagen.

The tissue specimens were inspected upon arrival and transverse sections of 5 mm were made, aiming to preserve both the base and the surface of the lesion. Two neighbouring biopsy specimens were placed in either 10% neutral buffered formalin or in tubes without fixative, the latter stored at −80 °C until DNA extraction. Blood samples were centrifuged at 2000 *g* for 10 min and the serum stored at −80 °C until analysis. Data for each animal were obtained from the Danish Central Cattle Database and included breed, gender, herd of origin, and dates of birth and of slaughter.

### Histopathology

The formalin fixed samples were routinely processed for histopathology, embedded in paraffin, sectioned at 3 μm, and stained with haematoxylin and eosin (H&E). Light microscopy was performed non-blinded for cases 1–50, while cases 51–100 were examined blinded to results of laboratory analysis (PCR and ELISA) by one researcher (JSA). Sections of a single case (Case #43) was additionally stained using periodic acid–Schiff (PAS), phosphotungstic acid haematoxylin (PTAH) and by the Masson’s trichrome method for connective tissue.

### Serology

Serum samples were tested for antibodies to *C. burnetii* using an indirect enzyme-linked immunosorbent assay (ELISA) (LSIVet Ruminant Q Fever Serum/Milk ELISA Kit, Laboratoire Service International) according to the manufacturer’s instructions. Briefly, serum was diluted 1:400 in dilution buffer and transferred to wells of ELISA plates coated with antigen (total volume 100 μL). The plates were incubated for 1 h at 37 °C followed by washing three times and incubation with 100 μL anti-ruminant IgG peroxidase conjugate for 1 h at 37 °C. After washing three times, wells were incubated with 100 μL tetramethylbenzidine substrate for 10 min at room temperature (around 22 °C) in the dark. Colour development was stopped by adding 100 μL 0.5 M H_2_SO_4_. Absorbance values were measured at 450 nm (OD_450_). Antibody reactivity was calculated using the sample to positive ratio (S/P) calculated as (Sample OD – Negative OD) / (Positive OD – Negative OD) × 100. The S/*P* values were categorised as negative (S/P ratio ≤40) or positive (S/P ratio > 40).

### Real-time PCR

DNA was extracted using the DNeasy Blood & Tissue Kit (Qiagen), according to the manufacturer’s instructions. Samples of approximately 4 mm^3^ in size were processed from the surface of vegetative changes. DNA samples were tested by a real-time PCR designed to detect the *C. burnetii*-specific IS*1111a* element [20] using specific primers and probe (Sense: 5′-CATCACATTGCCGCGTTTAC-3′, antisense: 5′-GGTTGGTCCCTCGACAACAT-3′, probe: FAM-AATCCCCAACAACACCTCCTTATTCCCAC-BHQ1). An inhibition control was used which utilizes the primers for the IS*1111a* element and a dedicated probe (YakimaYellow-ACATAATCTCTCCGACCCCACACTTCCATAC-BHQ1).

Reactions were performed using a 7500 Fast Real Time PCR system (Applied Biosystems) and consisted of 5 μL DNA template, 400 nM of each primer and 200 nM of each probe in 10 μL PerfeCTa Multiplex qPCR Supermix, uracil-N-glycosylase (2×) (New England Biolabs), with 0.4 μL Low Rox dye (Quanta BioSciences), 1 μL of inhibition control, and distilled water up to a total volume of 15 μL. An initial uracil DNA glycosylase (UDG) incubation for 5 min at 45 °C and denaturation/activation for 60 s at 95 °C was followed by 50 cycles of denaturation for 10 s at 95 °C, annealing/elongation for 30 s at 60 °C. Results were analysed with 7500 Fast System Software (Applied Biosystems) and scored negative when no amplicon was generated, or when the cycle threshold (Ct) value was >40; positive for Ct values ≤36, and inconclusive for Ct values between 36 and 40. When inhibition of the internal positive control was observed, samples were retested at a dilution 1 in 10.

### Fluorescence in situ hybridization (FISH)

Cases were selected (*n* = 10) that demonstrated the highest infection load (i.e. lowest Ct values on real-time PCR) for *C. burnetii,* and controls (*n* = 10) were randomly selected from those with negative serology and PCR results. Serial sections of 3 μm thickness were prepared and subjected to FISH examination, blinded to the test results. Two oligonucleotide probes targeting the 16S rRNA were used: Eub-338; 5′-GCTGCCTCCCGTAGGAGT-3′ for the entire domain Bacteria and S-S-C.burnetti-443; 5′-CTTGAGAATTTCTTCCCC-3′ for *C. burnetii* [21]. The oligonucleotide probes were labelled at the 5′ end with fluorescein isothiocyanate (FITC) (Eub-338 and S-S-C.burnetti-443), or Cy3 (Eub-338 and S-S-C.burnetti-443) (Eurofins MWG Operon). The FITC-labelled Eub-338 and the Cy3-labelled S-S-C.burnetti-443 probes were used in a double hybridization, whereas the two other probes were used individually.

Hybridization was performed at 45 °C with 40 μL of hybridization buffer (100 mM Tris [pH 7.2], 0.9 M NaCl, 0.1% sodium dodecyl sulfate) and 200 ng of each probe for 16 h in a Sequenza slide rack (Thermo Shandon). The sections were washed three times at 45 °C in hybridization buffer for 15 min and subsequently three times in washing solution (100 mM Tris [pH 7.2], 0.9 M NaCl). The sections were rinsed in water, air dried, and mounted in Vectashield (Vector Laboratories) for fluorescence microscopy. An Axioimager M1 fluorescence microscope, equipped with a 100-W HBO lamp and filter sets 43 and 38 were used to visualize Cy3 and FITC, respectively.

### Statistical analysis

Data were analysed in SAS version 9.4 (SAS Institute). ELISA and PCR results were considered to be ordinal variables and testing of associations between age and ELISA or PCR values were evaluated by Spearman’s correlation, linear regression and analysis of variance. Associations between gender and breed versus ELISA and PCR values were evaluated in contingency tables using the chi-square test. Associations between ELISA and PCR results were tested by Spearman’s correlation analysis and associations between ELISA and PCR results, categorized as test positive and test negative, were evaluated by Kappa estimation and McNemar’s test. Associations between type and number of heart valves affected was analysed in contingency tables using Fisher’s exact test.

## Results

The samples originated from dairy cattle, mainly Holsteins (83%), and most cases were female (96%). Mean ages of males and females were 20.5 months (SD 12.2 months) and 53.0 months (SD 23.0 months), respectively. The cases originated from 92 herds (two animals from each of eight herds and one animal from each of the remaining 84 herds). The right atrioventricular (tricuspid) valve was most frequently affected (58%), followed by the left atrioventricular (mitral) (31%), pulmonary (24%) and aortic valve (16%), as recorded by the meat inspection personnel at the abattoirs. A likely source of systemic infection was identified in 17 cases (peritonitis/traumatic reticuloperitonitis, *n* = 11; liver abscesses, *n* = 6). Other lesions consistent with bacteraemia, such as embolic pneumonia, nephritis or osteomyelitis, were reported in around 50% of the endocarditis cases.

**Fig. 1:**
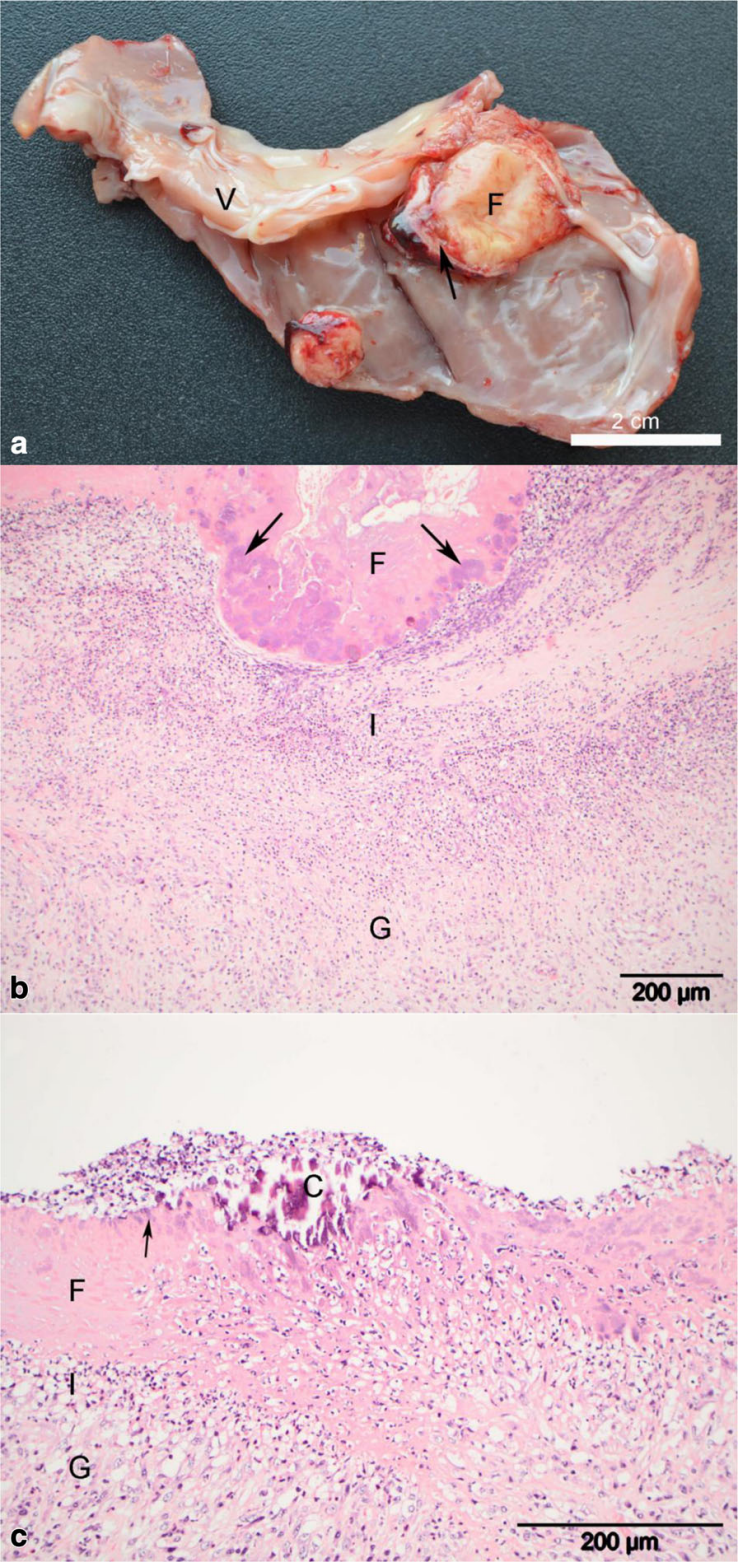
Chronic bovine fibrinous endocarditis. **a** gross pathology showing two areas of endocardial inflammation. The largest lesion, which is located in the valve (V), is cut through thus exposing a central core of fibrous tissue (F) that is externally covered by fibrin and clots (*arrow*). A minor mural lesion is similarly covered by fibrin and a clot; **b** + **c** Photomicrographs of endocardial lesions showing a core of granulation tissue (G) that is externally bordered by a zone of suppurative inflammation (I). The surface of the lesions is covered by fibrin (F) in which multiple bacterial colonies are embedded (*arrows*). **c** A focus of calcification (C) is present on the surface, which is also covered by a layer of neutrophils. Such calcified foci are consecutively embedded first in the fibrin and later in the granulation tissue as the lesion continues to expand by superficial opposition of fibrin and organisation into fibrous tissue from the base. (H&E)

**Fig 2:**
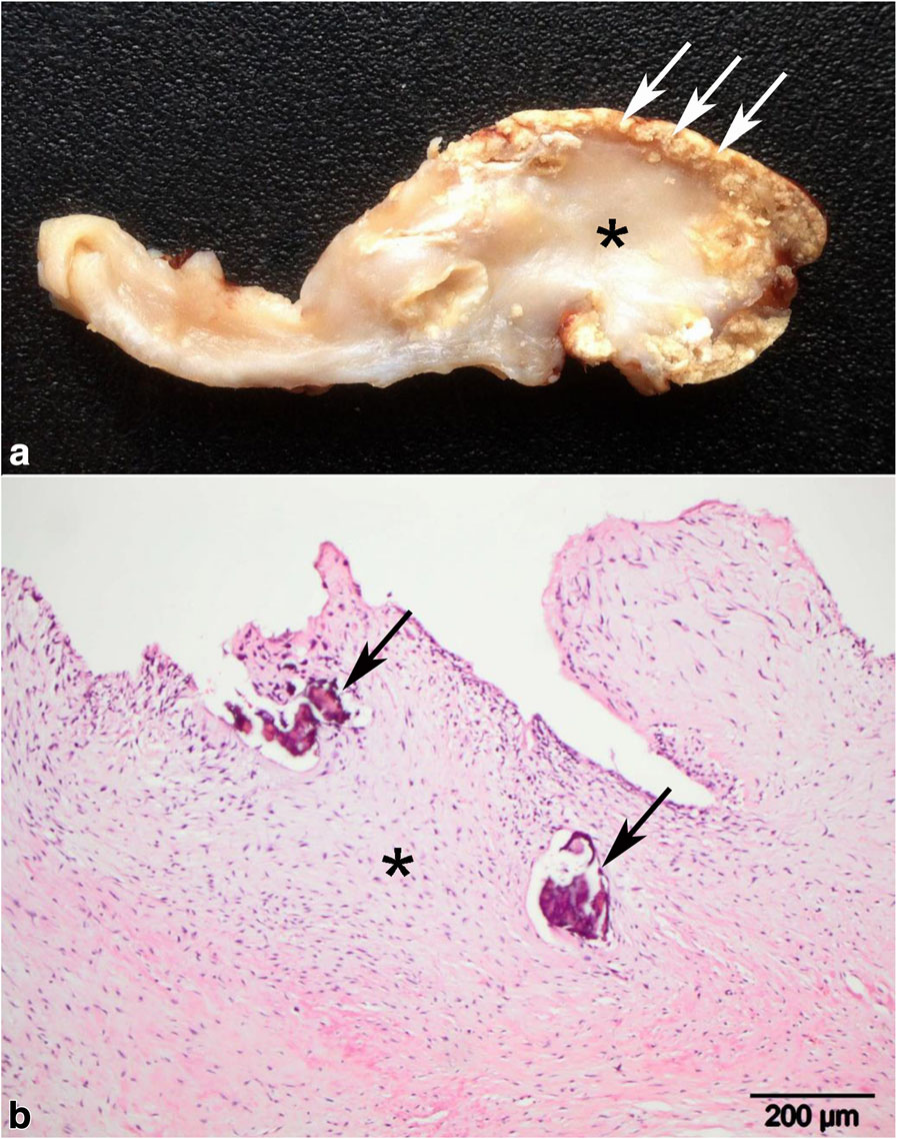
Chronic bovine fibrous calcified endocarditis. **a** Gross pathology showing extensive fibrosis and thickening of the stroma (*) and widespread superficial calcification (*arrows*). Formalin fixed specimen. **b** Histopathology of the lesion, showing extensive fibrosis (*) and areas of calcification (*arrows*). (H&E)

Most cases (*n* = 95) demonstrated chronic valvular vegetative endocarditis, affecting one or more valves, sometimes with adjacent mural lesions. Lesions were characterised by a core of pale fibrous tissue covered by significant amounts of fibrin and with a coagulum covering the surface (Fig. 1). Intralesional liquefaction was present in 27 cases. The lesions varied in size from <1 cm to several cm. One case had only acute/subacute lesions, which were characterized by haemorrhagic less extensive lesions without grossly visible granulation tissue. In other cases (*n* = 4), including Case #43, there was evidence of more chronic, organised lesions, characterized by extensive fibrosis and calcification (Fig. 2).

In terms of the histopathology findings, in general there was a core consisting of fibrous tissue, with inflamed granulation tissue at the periphery. This was covered by a zone of suppurative inflammation with overlying layers of fibrin containing basophilic bacterial colonies and neutrophils, either widely dispersed or as accumulations of bacteria and debris (Fig. 1). An acute/subacute case was characterized by intra-valvular haemorrhage, slight suppurative inflammation and endothelial ulceration covered by fibrin in some locations, while more established lesions were characterised by valvular fibrosis and intact endothelium. In some of the chronic cases (*n* = 21), calcification was present within the layers of fibrin, in the zone of intense inflammation and in the fibrous core. The most widespread calcification was observed in chronic fibrous cases with intact/healed endothelium. Osseous metaplasia was present in one case.

**Fig. 3:**
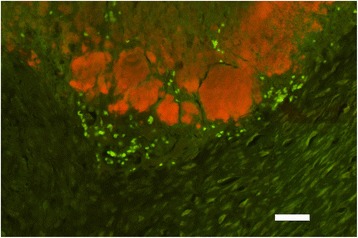
Detection of bacteria in chronic bovine fibrinous endocarditis by fluorescence in situ hybridization. Bacterial colonies (*red*) within fibrin layers of a suppurative endocardial lesion. Hybridization for the domain Bacteria (Cy3 labelled), bar = 40 μm

None of the cases examined for *C. burnetii* by FISH were found to be positive, by use of the FITC nor Cy3 labelled probes. Similar to the H&E stained sections bacterial colonies of varying seize were observed in the fibrin layers of the suppurative inflammation when hybridized with the probe for the domain Bacteria (Fig. 3) with a bacterial morphology varying from coccoid to small rod-like.

Serology revealed that 70% of the endocarditis cases were positive for antibodies against *C. burnetii*. PCR analysis of heart valve lesions identified 25% as being positive, 52% negative and 23% were inconclusive. Ct values for the positive samples ranged from 33 to 36 (i.e. low copy number), except for one animal, a 7-year-old Jersey cow (Case #43) that demonstrated a Ct value of 13.7 in the aortic valve, and an ELISA S/*P* value of 82%.

**Fig. 4:**
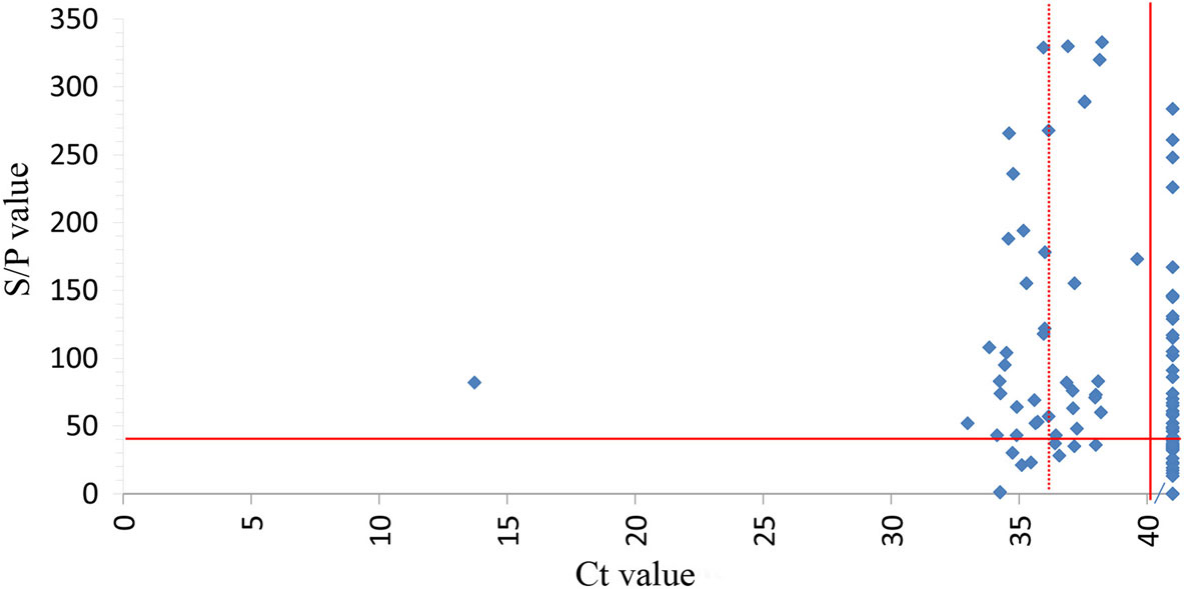
Scatter plot of *Coxiella burnetii* DNA in bovine endocarditis lesions and corresponding serology results. ELISA S/*P* values >40 are considered to be positive, those ≤40 are considered to be negative. PCR Ct values ≤36 are considered to be positive, those between 36 and 40 are inconclusive and those >40 are considered negative. Samples with no amplicon present (i.e. below the detection limit) were assigned a nominal Ct value of 41. Cut-off values are indicated by *red lines* and inconclusive by a *dotted line*

Figure 4 is a scatter plot of the relations between ELISA and PCR values. Regression analysis showed there was no statistical significant association between ELISA and PCR values (*P* = 0.28), and when these were converted to categorical data (i.e. test positive vs. negative), estimation of Kappa (0.12) with associated McNemar’s test also failed to show any significant agreement. However, Spearman’s correlation analysis showed a weak association (*R*_S_ = −0.225, *R*_S_^2^ = 0.05, *P* = 0.025). Spearman’s correlation, regression analysis and analysis of variance showed no significant associations between age and ELISA or PCR results. There was no significant relationship between gender or breed versus ELISA or PCR results.

## Discussion

In humans, detection of *C. burnetii* by culture, PCR or immunohistochemistry of a cardiac valve is required for making a definitive diagnosis of Q fever endocarditis [22]. However, in a number of PCR- or culture-positive cases, there are no signs of inflammation [23], highlighting the challenges associated with making this diagnosis. Fulminant Q fever valvular endocarditis in humans is characterized by inflammation of either acute or chronic active nature, dominated by focal or diffuse infiltrations by lymphocytes, histiocytes and occasional plasma cells. Microabscesses and necrosis are present in some cases and the surface is covered by fibrinous vegetations and leukocytic infiltrations, while the valvular stroma shows extensive fibrosis and calcification. Macrophages containing intracellular bacteria may be demonstrated in the vegetations by use of special stains or immunohistochemistry [24–26].

The bovine endocarditis lesions examined in the present study were similar to those described in human Q fever endocarditis, although they were probably more advanced in nature as bovine endocarditis may remain undiagnosed for a long period of time due to lack obvious clinical signs until severely compromised valvular function. Despite the similarities in histopathology, the association between the development and progression of endocarditis and *C. burnetii* infection is not clear. Huge numbers of bacteria with morphology and staining abilities consistent with *T. pyogenes* were commonly visualised within the endocarditis lesions thus showing that development and progression of the lesion may be associated with another primary bacterial infection rather than with *C. burnetii*. Also around 50% of the cattle had lesions consistent with pyaemia, a condition likely to be the main reason for many animals to develop endocarditis. Bacterial culturing was not performed as the present study focused on detection of *C. burnetii* only and because the culturable bacterial flora of bovine endocarditis has been examined thoroughly in several studies [10, 12, 15]. The PCR results indicated that even in positive samples, the copy number (indicative of infection ‘load’) was low in all but one animal (Case #43). It is therefore tempting to speculate that *C. burnetii* contributed to the endocarditis in most cases as a co-inhabitant of a primary *T. pyogenes* pyaemia-associated endocarditis.

A secondary role of *C. burnetii* in the development of bovine endocarditis is also supported by the negative FISH results. The FISH technique only detects bacteria in active growth, so in addition to a low infection level, negative FISH results may also indicate presence of only inactive bacteria. Furthermore, *C. burnetii* is difficult to visualize due its small size. So unless present in bacteria-loaded macrophages, which indicates intracellular multiplication, detection of *C. burnetii* by FISH is challenging. As macrophages loaded with *C. burnetii* were absent, a major role of *C. burnetii* in the development and progression of bovine endocarditis seems less likely.

In the specimen with the most conclusively positive PCR result (Case #43), the pathology was dominated by extensive fibrosis and calcification. Such lesions have been reported in cases of human Q fever endocarditis and more commonly occur in association with *C. burnetii* than in lesions caused by another aetiology [26]. Whether extensive fibrosis and calcification is associated with *C. burnetii* in cattle remains to be studied. This type of lesion may represent the end-stage of healing of a previous vegetative endocarditis. Calcification within the fibrin layers, at the borders between fibrin and granulation tissue and within the granulation tissue was found in many of the examined cases and suggests that calcification develops within the fibrin layer and gradually becomes embedded in the connective tissue core, during re-organisation of the fibrinous mass from its base. Such calcification could persist for prolonged periods in the fibrotic core, either as inert calcification or within granulomas, if associated with a foreign body reaction. Examination of other cases is needed to determine whether extensive fibrosis and calcification are associated with *C. burnetii* endocarditis, or if the presence of *C. burnetii* in such cases simply reflects that the animal was at risk of *C. burnetii* colonisation due to a pre-existing long lasting valvulopathy.

Most of the cattle examined (70%) were seropositive for antibody reactivity to *C. burnetii* by ELISA. In Denmark, recent studies have shown that 59–79% of bulk milk samples of dairy cattle herds are antibody positive [27, 28] thus demonstrating that Danish dairy cattle are commonly exposed to this organism. The presence of *C. burnetii* DNA in 25% of the endocarditis valves shows that cattle may experience coxiellaemia following exposure, which potentially might lead to colonisation of the cardiac valves when other predisposing conditions are present. This case series focused on detection of *C. burnetii* in cases of endocarditis and did not include normal heart valves, so a possible presence of *C. burnetii* in normal heart valves remains uncertain. It is therefore not possible to determine the role of *C. burnetii* in the initiation of endocardial inflammation as only grossly affected valves were included in the study. A case-control study should be conducted to obtain more information on the role of *C. burnetii* in the development of bovine valvular endocarditis. However, as valvulopathy, including pre-existing valvular endocarditis, is a recognized risk factor for developing *C. burnetii* associated endocarditis in humans, cattle with such lesions most likely have a higher risk for *C. burnetii* valvular colonisation than normal cattle. In cases of vegetative endocarditis, the continued apposition of fibrin probably gives *C. burnetii* bacteria passing the valves in case of coxiellaemia a greater chance of being trapped and subsequently infecting the valve than in animals without pre-existing valvulopathy.

Because the statistical analysis showed a weak correlation between the ELISA and PCR values (*P* = 0.025), and regression analysis and the estimation of Kappa with the associated McNemar’s test did not show any significant agreement between PCR and ELISA results, we conclude that the relationship between the serology and PCR data is not conclusive. Given the high seroprevalence in the cattle population and the relatively low prevalence of endocarditis, it is not likely that serology can be used as part of the diagnostic approach for Q fever endocarditis. The cow with high infection load (Case #43) illustrates this point as it had a relatively low ELISA S/*P* value. Serology and PCR have been used to study cows excreting *C. burnetii* in milk or vaginal discharge, but the association is usually weak [29–31], although many cows that persistently excrete *C. burnetii* organisms in the milk often show high antibody titres [32].

## Conclusions

The findings indicate the presence of *C. burnetii* DNA in a proportion of heart valve lesions in cattle. The exact role of *C. burnetii* in the development and progression of lesions remains unknown as most cases were also infected by huge numbers of *T. pyogenes*; a bacterium known to be associated with severe endocarditis in cattle. *C. burnetii* PCR positive and negative cases could not be distinguished pathologically, but it is noteworthy that the cow with the highest infection level had extensive valvular fibrosis, widespread calcification and an intact endothelium thus being morphologically unique in this case series. Cattle affected with vegetative endocarditis are usually condemned as unfit for human consumption and therefore are unlikely to pose a zoonotic risk to humans. There is a need for more research into the clinical consequences of Q fever in ruminants, beyond those focusing on reproductive disease. The spectrum of clinical signs seen in the human infection should be taken into consideration, when assessing the possible health implications in livestock. In particular, the role of *C. burnetii* in bovine endocarditis merits further investigation, including examination for its possible presence in normal heart valves.

## Abbreviations

*C. burnetii*: *Coxiella burnetii*
Ct: Cycle threshold
ELISA: Enzyme-linked immunosorbent assay
H&E: Haematoxylin and eosin
PCR: Polymerase chain reaction
S/P: Sample to positive ratio
SD: Standard deviation
*T. pyogenes*: *Trueperella pyogenes*
